# Clinical, Hereditary, and Lifestyle Factors Associated with Cardiovascular Disease in Adults with Type 2 Diabetes in Saudi Arabia: A Cross-Sectional Study

**DOI:** 10.3390/diagnostics16213581

**Published:** 2026-10-29

**Authors:** Mohammad A. Jareebi

**Affiliations:** Department of Family and Community Medicine, College of Medicine, Jazan University, Jazan 45142, Saudi Arabia; mjareebi@jazanu.edu.sa

**Keywords:** diabetes mellitus, cardiovascular disease, hypertension, dyslipidaemia, family history, whole grains, Saudi Arabia

## Abstract

**Background/Objectives**: Diabetes mellitus and cardiovascular disease (CVD) constitute a dual epidemic in Saudi Arabia. This study examined the associations of cardiometabolic conditions, behavioural risk factors, and family history with cardiovascular disease among adults with type 2 diabetes mellitus (T2DM). **Methods**: An analytical cross-sectional study was conducted on 423 adults with self-reported physician-diagnosed T2DM drawn from a community-based survey in the Jazan region, Saudi Arabia. Sociodemographic, anthropometric, behavioural, dietary, and clinical variables were assessed using a validated WHO STEPwise-based questionnaire. CVD status was determined through self-reported physician diagnosis. Bivariate analyses and multivariable logistic regression with Firth penalisation were used to identify factors associated with CVD, with a reduced model as a sensitivity analysis. **Results**: Participants had a mean age of 48 ± 15 years; 60% were male, and 98% were Saudi nationals. The prevalence of self-reported CVD was 9.7%. Family history of CVD showed the strongest association (OR = 14.57; 95% CI: 5.88–40.90; *p* < 0.001), followed by diagnosed dyslipidaemia (OR = 5.19; 95% CI: 1.91–14.22; *p* = 0.001) and diagnosed hypertension (OR = 4.71; 95% CI: 1.86–13.16; *p* < 0.001). Regular whole-grain consumption (OR = 0.30; 95% CI: 0.11–0.75; *p* = 0.010) and a monthly income of 5000–9999 SAR (OR = 0.23; 95% CI: 0.05–0.89; *p* = 0.032) were associated with lower odds of CVD; all five associations persisted in a reduced model. Body mass index, physical inactivity, and smoking were not significantly associated with CVD after adjustment. **Conclusions**: In this exploratory analysis of adults with T2DM, CVD was strongly associated with family history of CVD, hypertension, and dyslipidaemia, and inversely associated with whole-grain intake. Given the cross-sectional design, self-reported measures, and wide confidence intervals, these associations do not establish causality and require prospective confirmation; family history of CVD may nonetheless help prioritise patients for cardiovascular risk assessment.

## 1. Introduction

Diabetes mellitus has reached pandemic proportions. The 11th edition of the International Diabetes Federation (IDF) Diabetes Atlas estimates that 589 million adults aged 20–79 years are living with diabetes worldwide, a figure projected to rise to 853 million by 2050 [1]. Approximately 252 million of these individuals (42.8%) are unaware of their condition [2]. Type 2 diabetes mellitus (T2DM) accounts for roughly 90% of all diabetes and is associated with an 84% higher risk of incident heart failure compared with people without diabetes [1]. Cardiovascular disease (CVD) remains the leading cause of mortality globally, accounting for approximately 32% of all deaths [3]. The global prevalence of CVD was estimated at 598 million cases in 2025, with 20.5 million annual deaths; both figures are projected to rise to 1.14 billion cases and 35.6 million deaths by 2050, driven largely by hypertension, dyslipidaemia, obesity, and diabetes [4]. A recent prospective cohort study reported that diabetes was independently associated with a 2.37-fold higher risk of incident CVD events (95% CI 1.38–4.08) [5]. These figures underscore why the co-occurrence of diabetes and CVD is now described as a dual epidemic that demands targeted, population-specific research into its determinants.

Saudi Arabia exemplifies this dual burden. National surveys estimate the adult prevalence of diabetes at 23–24%, among the highest in the world [6,7]. Concurrently, non-communicable diseases account for 73% of all deaths in the Kingdom, with CVD responsible for 37% [8]. The projected ten-year economic burden of atherosclerotic CVD in Saudi Arabia exceeds 106 billion Saudi Riyals (SAR) [8]. Obesity, a key intermediate risk factor, affects 20–39% of Saudi adults [9], and approximately 52% of patients with diabetes in the Kingdom die from cardiovascular causes [10]. Despite these alarming figures, granular patient-level data quantifying the independent contributions of clinical and lifestyle determinants of CVD among Saudi adults with diabetes remain scarce.

The literature consistently identifies two broad clusters of determinants that interact to amplify cardiovascular risk in people with diabetes. Diabetes itself is associated with a substantially higher risk of fatal coronary heart disease, with a greater relative excess in women than in men (relative risk 3.50, 95% CI 2.70–4.53 vs. 2.06, 95% CI 1.81–2.34) [11]. Dyslipidaemia further compounds risk, as diabetic individuals with adverse lipid profiles have a 2.19-fold higher risk of CVD events (95% CI 1.29–3.70) [5], and a 2025 systematic review reported a 56% increase in cardiovascular events associated with hyperlipidaemia in diabetic populations (relative risk 1.56, 95% CI 1.34–1.82) [12]. Family history is also a powerful determinant: a positive family history of diabetes is associated with a 54% increase (95% CI 41–67%) in the risk of advanced cardiovascular–kidney–metabolic syndrome, and a family history of ischaemic heart disease with a 147% increase (95% CI 123–173%) [13]. Behavioural and lifestyle factors are equally consequential. Obesity is associated with a two- to three-fold higher risk of cardiovascular mortality [14], and in Saudi Arabia, hypertension (67.6%), diabetes (60.7%), and hypercholesterolaemia (51.3%) are the most common obesity-related comorbidities, with risk increasing across BMI categories [9]. Physical inactivity accounts for approximately 10% of stroke and 7% of heart failure among people with diabetes [15], smoking increases overall CVD risk by approximately 50% in people with diabetes [16], and each additional unfavourable lifestyle behaviour increases incident CVD risk by 2–9% [17].

Despite this evidence, patient-level data on the joint associations of clinical, hereditary, and lifestyle factors with CVD in adults with diabetes in Saudi Arabia remain limited. Earlier analyses of the same community survey examined the correlates of hypertension [18] and of diabetes [19] but did not examine CVD among participants with diabetes. Identifying which readily obtainable characteristics, such as family history and diagnosed comorbidities, are most strongly associated with CVD in this group could help clinicians prioritise patients for cardiovascular assessment. This cross-sectional study therefore aims to estimate the prevalence of self-reported CVD among adults with T2DM in Saudi Arabia and to examine its associations with clinical, hereditary, and lifestyle factors.

## 2. Materials and Methods

### 2.1. Study Design, Setting, and Population

This cross-sectional analytical study examined clinical, hereditary, and lifestyle factors associated with CVD among adults with T2DM in the Jazan region, southwestern Saudi Arabia. It was conducted as a secondary analysis of a community-based health survey carried out in May–June 2024, whose design is described in the parent publications [18,19]. A multistage approach was used: the region was stratified into urban and rural areas, community settings such as universities, healthcare centres, and community centres were purposively selected within each stratum, and consenting adults were recruited by convenience sampling at each site. The survey enrolled 3411 adults, of whom 3312 were Saudi nationals; an analysis restricted to the Saudi participants has been reported [18]. The present analysis included the 423 participants aged 18 years or older who reported a physician diagnosis of T2DM (414 Saudi and 9 non-Saudi nationals); participants with type 1 diabetes had been excluded from the parent dataset [19], and one participant younger than 18 years was excluded. The sociodemographic, anthropometric, and dietary characteristics of participants with diabetes and the correlates of diabetes itself were reported previously [19]; CVD among participants with diabetes has not been analysed before.

### 2.2. Data Collection

Data were collected using a structured, pretested, Arabic-language questionnaire administered under the supervision of trained medical and public-health students, as described in detail in the parent publications [18,19]. Briefly, the questionnaire was based on the WHO STEPwise Approach to Surveillance (STEPS) tool [20]; physical activity was assessed using the Arabic version of the International Physical Activity Questionnaire (IPAQ) [21,22]; and dietary intake was assessed using validated nutritional-assessment items [23]. Chronic conditions were ascertained by asking whether the participant had ever been diagnosed with each condition, answered yes or no separately for diabetes, hypertension, CVD, and dyslipidaemia. Height and weight were self-reported. The full questionnaire is available in the parent publication [19].

### 2.3. Study Variables

Outcome. The primary outcome was self-reported, physician-diagnosed CVD, recorded as a single yes/no item. The item referred to CVD in general and did not distinguish coronary heart disease, stroke, heart failure, or other cardiovascular conditions.

Exposures. Sociodemographic variables included age, sex, nationality, education, employment, marital status, monthly household income, residence, housing type, and family size. Body mass index (BMI, kg/m^2^) was calculated from self-reported height and weight and classified using WHO cut-offs [24]. Behavioural variables included physical activity (assessed by IPAQ and categorised as no weekly activity; <30 min of moderate/vigorous activity 5 days/week; or ≥30 min of moderate/vigorous activity 5 days/week), smoking status (never, current, ex-smoker; participants reporting only second-hand smoke exposure were classified as never smokers), and sleep duration. Dietary variables included regular whole-grain consumption, intake of ≥5 servings of fruits and vegetables per day, choice of low-fat meats, choice of low-fat dairy, and avoidance of high-sugar foods. Clinical and hereditary variables included diagnosed hypertension, diagnosed dyslipidaemia, family history of diabetes, family history of hypertension, and family history of CVD (each in first-degree relatives).

### 2.4. Statistical Analysis

Analyses were performed in R version 4.3.3 (R Foundation for Statistical Computing, Vienna, Austria), using the logistf package for penalised regression. Continuous variables are summarised as means with standard deviations; categorical variables are summarised as frequencies and percentages. Univariate associations between each candidate variable and CVD diagnosis were assessed using the chi-square test or Fisher’s exact test for categorical variables and the independent-samples *t*-test for continuous variables. A two-sided *p*-value < 0.05 was considered statistically significant.

Two multivariable logistic regression models were fitted with CVD diagnosis as the dependent variable. Candidate variables were specified a priori from the survey content and entered simultaneously without data-driven selection: age, sex, nationality, education, employment, marital status, monthly income, residence, housing type, family size, BMI (continuous), physical activity, smoking status, the five dietary variables, family history of diabetes, hypertension, and CVD, and diagnosed hypertension and dyslipidaemia, giving 29 parameters for 41 events. All candidate variables were retained rather than selected statistically, because data-driven selection with few events produces unstable models and biased estimates; the full model was therefore treated as an exploratory description of adjusted associations, not as a prediction model. Sleep duration was summarised descriptively and was not entered in the models. The first model used standard maximum-likelihood estimation. Because the number of events was small relative to the number of parameters, a Firth penalised-likelihood logistic regression model was fitted as the primary multivariable model to reduce small-sample bias [25,26]. As penalisation does not by itself prevent overfitting, a sensitivity analysis fitted a reduced model restricted to established cardiovascular risk factors (age, sex, BMI, smoking status, physical activity, diagnosed hypertension, diagnosed dyslipidaemia, and family history of CVD; 10 parameters), to which whole-grain consumption and income were added one at a time. Participants with incomplete responses to key variables had been excluded from the parent dataset [19]; the number excluded at that stage was not recorded and therefore cannot be reported, and the analytic dataset contained no missing values, so all 423 participants were included in every model. Results are presented as odds ratios (ORs) with 95% confidence intervals (CIs), using profile likelihood CIs for the standard model and profile penalised-likelihood CIs with penalised likelihood ratio *p*-values for the Firth model. Model fit was summarised using Tjur’s coefficient of discrimination (Tjur R^2^).

### 2.5. Ethical Considerations

Ethical approval was granted by the Standing Committee for Scientific Research at Jazan University (IRB approval No. REC-45/05/848, dated 26 November 2023). All participants provided written informed consent; participation was voluntary, and the study was conducted in accordance with the principles of the Declaration of Helsinki.

### 2.6. Use of Generative Artificial Intelligence

ChatGPT (GPT-5.6, OpenAI, San Francisco, CA, USA) was used solely for language editing, proofreading, and improvement of English grammar and clarity. The authors reviewed and approved all AI-assisted revisions and take full responsibility for the content of the manuscript.

## 3. Results

### 3.1. Sociodemographic Characteristics

A total of 423 diabetic patients were included in the study (Table 1). The mean age was 48 years (SD ± 15; range 18–90). The majority were male (60%), Saudi nationals (98%), and Arabic-speaking (100%). Educational attainment was evenly distributed, with 53% holding a bachelor’s degree or higher and 47% having completed high school or below. Most participants were employed (62%) and married (73%). Approximately two-thirds resided in rural areas (63%), and nearly half owned a villa (45%). Mean family size was 7.3 persons (SD ± 2.9). Monthly income was fairly distributed across brackets, with the largest proportion earning ≥15,000 SAR (30%) and the smallest earning 5000–9999 SAR (17%) (Table 1).

### 3.2. Habitual and Health-Related Characteristics

The mean BMI was 27.0 kg/m^2^ (SD ± 5.4). The majority of participants were classified as overweight (38%) or obese (26%), while 32% were of normal weight and 4% were underweight. Physical inactivity was highly prevalent, with 43% of participants reporting no weekly physical activity; only 18% met the recommended minimum of at least 30 min of moderate or vigorous activity five times per week. Most participants (78%) had never smoked, 12% were current smokers, and 10% were ex-smokers. Mean sleep duration was 7.3 h per night (SD ± 1.8). Family history of diabetes mellitus was reported by 86% of participants, family history of hypertension by 58%, and family history of CVD by 20%. Comorbid hypertension was present in 48%, dyslipidemia in 10%, and CVD in 10% of the sample (Table 2).

### 3.3. Nutritional Characteristics

Less than half of participants consumed whole grain products (47%) or met the recommended minimum of five servings of fruits and vegetables per day (26%). The majority chose low-fat meats (60%) but fewer selected low-fat products generally (38%). Just under half reported avoiding foods high in sugar (46%) (Table 3).

### 3.4. Bivariate Associations with CVD Diagnosis

CVD was diagnosed in 41 of 423 participants (10%). Univariate analyses identified six variables significantly associated with CVD at *p* < 0.05. Whole grain consumption was significantly lower among CVD-positive patients (29% vs. 49%; *p* = 0.025). Smoking history differed significantly between groups (*p* = 0.048), with ex-smokers overrepresented among CVD cases (20% vs. 9%) and current smokers underrepresented (5% vs. 13%). Family history of hypertension was more prevalent among CVD patients (78% vs. 56%; *p* = 0.011). Family history of CVD showed the strongest univariate association, present in 68% of CVD cases compared to only 15% of non-CVD patients (*p* < 0.001). Comorbid dyslipidemia (27% vs. 9%; *p* < 0.001) and hypertension (83% vs. 44%; *p* < 0.001) were also significantly more prevalent among CVD cases. Age, sex, BMI, BMI category, physical activity, education, income, and other dietary variables did not reach statistical significance at the univariate level (Table 4).

### 3.5. Multivariable Analysis of Factors Associated with CVD

**Figure 1:**
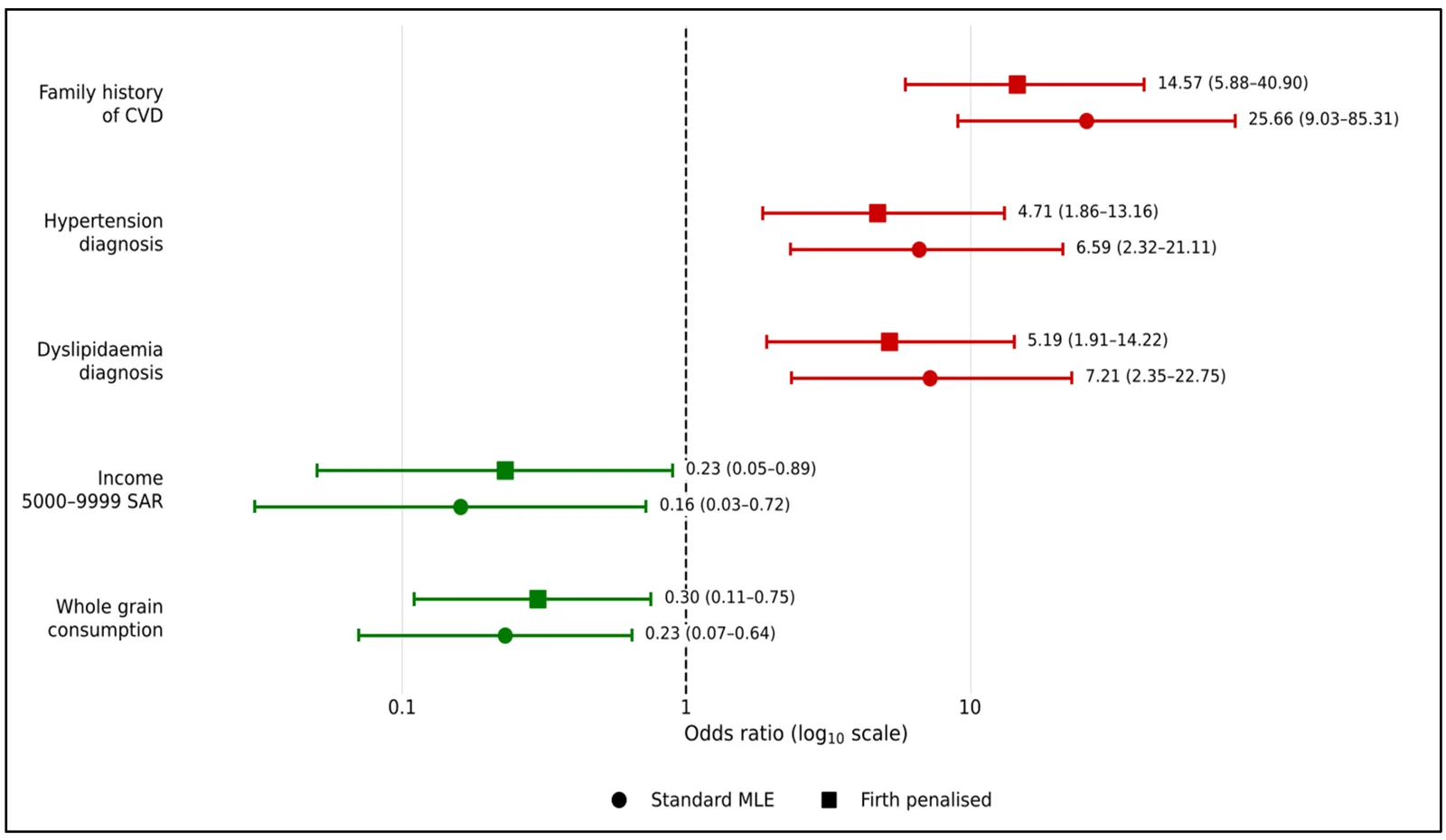
Forest plot of variables significantly associated with cardiovascular disease in the full models among participants with type 2 diabetes mellitus (n = 423). Odds ratios (ORs) are displayed on a log_10_ scale with 95% confidence intervals. Circles represent standard maximum likelihood estimates; squares represent Firth penalised likelihood estimates. Green denotes inverse associations (OR < 1); red denotes positive associations (OR > 1). The dashed vertical line indicates OR = 1.0 (null effect). All displayed variables were significant at *p* < 0.05 in both models.

Two multivariable logistic regression models including all 29 parameters were fitted: a standard maximum likelihood model and a Firth penalised likelihood model (Table 5 and Figure 1). In the standard model, family history of CVD (OR = 25.66, 95% CI: 9.03–85.31, *p* < 0.001), diagnosed dyslipidemia (OR = 7.21, 95% CI: 2.35–22.75, *p* < 0.001), and diagnosed hypertension (OR = 6.59, 95% CI: 2.32–21.11, *p* < 0.001) were associated with higher odds of CVD, whereas whole grain consumption (OR = 0.23, 95% CI: 0.07–0.64, *p* = 0.007) and income of 5000–9999 SAR compared with less than 5000 SAR (OR = 0.16, 95% CI: 0.03–0.72, *p* = 0.024) were associated with lower odds.

In the Firth penalised model, the same five variables remained statistically significant. Family history of CVD showed the strongest association (OR = 14.57, 95% CI: 5.88–40.90, *p* < 0.001), followed by diagnosed dyslipidemia (OR = 5.19, 95% CI: 1.91–14.22, *p* = 0.001) and diagnosed hypertension (OR = 4.71, 95% CI: 1.86–13.16, *p* < 0.001). Penalisation attenuated these three estimates by 43%, 28%, and 28%, respectively, relative to the standard model. Whole grain consumption (OR = 0.30, 95% CI: 0.11–0.75, *p* = 0.010) and income of 5000–9999 SAR (OR = 0.23, 95% CI: 0.05–0.89, *p* = 0.032) remained associated with lower odds of CVD. No other variable was significantly associated with CVD in either model (Table 5).

### 3.6. Sensitivity Analyses

In the reduced model restricted to established cardiovascular risk factors (10 parameters; Table 6), family history of CVD (OR = 14.04, 95% CI: 6.51–32.41), diagnosed hypertension (OR = 6.41, 95% CI: 2.71–17.02), and diagnosed dyslipidaemia (OR = 3.17, 95% CI: 1.23–7.89) remained associated with CVD in the Firth model. When added to this model one at a time, whole-grain consumption (OR = 0.39, 95% CI: 0.16–0.87, *p* = 0.021) and income of 5000–9999 SAR (OR = 0.22, 95% CI: 0.05–0.81, *p* = 0.021) retained their associations.

## 4. Discussion

### 4.1. Summary of Key Findings

This cross-sectional study of 423 adults with T2DM identified five factors associated with prevalent self-reported CVD. Family history of CVD showed the strongest association (OR 14.57), followed by diagnosed dyslipidaemia (OR 5.19) and diagnosed hypertension (OR 4.71). Regular whole-grain consumption (OR 0.30) and a monthly household income of 5000–9999 SAR were associated with lower odds of CVD compared with the reference groups, and all five associations were consistent in a reduced model restricted to established cardiovascular risk factors. Physical inactivity, smoking, and BMI were not significantly associated with CVD in the adjusted model. Given the small number of events relative to model parameters, these estimates are exploratory and imprecise, as reflected in their wide confidence intervals.

### 4.2. Clinical and Hereditary Factors

#### 4.2.1. Family History of Cardiovascular Disease

Family history of CVD showed the strongest association with prevalent CVD (adjusted OR approximately 15), but this estimate warrants caution. Its confidence interval was wide, reflecting the small number of events, and because both family history and CVD were self-reported, participants with CVD may recall or report affected relatives more completely, which would inflate the association. The direction of the association agrees with population-scale evidence: in 57,340 Korean adults, a family history of ischaemic heart disease was associated with a 147% higher risk of advanced cardiovascular–kidney–metabolic syndrome [13]. Self-reported family history and polygenic risk scores appear to capture overlapping but distinct components of inherited risk [27], but genetic susceptibility was not measured here, and the larger estimate in this sample may also reflect shared household behaviours or reporting bias. Family history of diabetes and of hypertension were not associated with CVD after adjustment, possibly because their effects operate through diabetes itself and through the diagnosed hypertension and dyslipidaemia already in the model, although this was not tested. Because it is simple to obtain, family history of CVD may help flag patients with diabetes for closer cardiovascular assessment, consistent with the 2025 Saudi Heart Association guidelines [28], but its value for this purpose would need to be evaluated prospectively.

#### 4.2.2. Hypertension

Diagnosed hypertension was the most prevalent comorbidity (48%) and was strongly associated with prevalent CVD (adjusted OR approximately 4.7). This finding is consistent with a recent analysis of the China Health and Retirement Longitudinal Study, which reported a supra-additive interaction between hypertension and diabetes for incident CVD [29]. In a Saudi setting, Alreshidi et al. reported a stroke incidence of 6% among patients with both diabetes and hypertension, compared with 4% among those with hypertension alone [30]. The persistence of this association after adjustment for dyslipidaemia, family history, and lifestyle factors is consistent with the established importance of blood-pressure control. The American Diabetes Association 2025 Standards of Care recommend a blood-pressure target below 130/80 mmHg for most diabetic patients [31], yet national Saudi data indicate that screening uptake and treatment coverage remain suboptimal [32].

#### 4.2.3. Dyslipidaemia

Diagnosed dyslipidaemia was associated with approximately five-fold higher odds of CVD despite being recorded in only 10% of participants. Atherogenic diabetic dyslipidaemia, characterised by elevated triglycerides, reduced HDL cholesterol, and small, dense LDL particles, poses a residual risk that often persists despite statin therapy, although triglyceride-lowering therapy has been shown to reduce both cardiovascular events and cardiovascular mortality in diabetic populations [33]. The 2024 European Society of Cardiology Cardiovascular Round Table emphasised individualised treatment intensification with ezetimibe, PCSK9 inhibitors, and/or icosapent ethyl [34]. The combination of a large effect estimate and a low recorded prevalence of dyslipidaemia may indicate that lipid abnormalities are under-diagnosed in this population, a pattern previously observed in Saudi diabetic cohorts [35]; because lipid levels were not measured, this explanation remains a hypothesis.

### 4.3. Dietary and Lifestyle Factors

#### 4.3.1. Whole-Grain Consumption

Of the dietary variables examined, only whole-grain consumption was associated with CVD after adjustment: regular consumers had approximately 70% lower odds of reporting CVD. This association should not be interpreted as a protective effect. Diet and CVD status were recorded at the same time, so dietary habits may have changed after diagnosis, and residual confounding by other health-related behaviours or socioeconomic factors is possible. The direction is nonetheless concordant with a burden-of-proof analysis of 27 prospective cohort studies, which estimated a 36.9% reduction in ischaemic heart disease risk and a 21.8% reduction in stroke risk associated with whole-grain intake [36], and with trial evidence in healthy adults that wholemeal foods favourably modify cardiovascular risk factors [37]. Whether increasing whole-grain intake lowers cardiovascular risk in adults with diabetes in Saudi Arabia, for whom barley, oats, and brown rice are culturally familiar options, requires prospective or interventional evaluation.

#### 4.3.2. Null Findings for Physical Activity, Smoking, and BMI

Physical inactivity, smoking status, and BMI were not significantly associated with CVD after adjustment. These null results do not show that these factors lack cardiovascular relevance: each was measured with a single self-reported item, the small number of events limited power, and cardiovascular benefits of activity in T2DM may require levels above those captured here [38]. Reverse causation may also contribute, as ex-smokers were more frequent among CVD cases (20% vs. 9%), consistent with cessation after diagnosis [39]. For BMI, part of any effect may operate through hypertension and dyslipidaemia, which were in the model, and self-reported anthropometry adds measurement error [40]; the obesity paradox described in diabetic populations [41] was not examined here.

#### 4.3.3. Sociodemographic Factors and Income

The lower odds of CVD in the 5000–9999 SAR stratum, without a consistent gradient across the other income groups, should be interpreted as exploratory, because only four CVD cases occurred in this stratum. Socioeconomic position can influence cardiovascular outcomes through healthcare access, health literacy, and diet, and a multicentre Gulf study reported greater coronary artery disease severity at higher income [42]; however, none of these pathways were measured here, and a chance finding cannot be excluded.

### 4.4. Implications for Clinical Practice and Public Health

These findings have several practical implications, provided they are confirmed prospectively. First, the strong association between family history of CVD and prevalent CVD supports documenting family history as part of cardiovascular risk assessment in patients with diabetes, in line with the 2025 National Heart Center/Saudi Heart Association recommendations [28]; its value as a screening question for undetected CVD would need to be evaluated against clinical assessment. Second, the associations with hypertension and dyslipidaemia, which affected approximately half and one-tenth of the sample, respectively, reinforce guideline-directed blood pressure and lipid management. Integrated care that addresses diabetes, hypertension, and dyslipidaemia together is recommended by the American Heart Association [43] and could be adapted to Saudi primary-care settings, where comprehensive cardiovascular screening currently reaches only a small proportion of the population [32]. Third, the inverse association with whole-grain intake identifies a culturally familiar dietary target whose cardiovascular benefit in this population should be tested in prospective or intervention studies.

### 4.5. Strengths and Limitations

This study has several strengths. To our knowledge, it is the first to simultaneously examine clinical, hereditary, and lifestyle factors associated with CVD in adults with T2DM in Saudi Arabia within a single multivariable model. The analysis drew on a community-based sample of 423 adults with diabetes. Data collection used a standardised WHO STEPS-based questionnaire and the validated Arabic version of IPAQ. Methodologically, the use of Firth penalised-likelihood logistic regression reduced small-sample bias in the effect estimates [26], and the main associations were consistent in a reduced model.

Despite these strengths, several limitations should be acknowledged. The cross-sectional design and self-reported outcome limit causal inference, limit clinical interpretation, and raise the possibility of reverse causation, particularly for behavioural and dietary exposures that may have changed after a CVD diagnosis. Self-reported CVD, hypertension, dyslipidaemia, and family history are subject to recall and misclassification bias; recall of affected relatives may also be more complete among participants with CVD, which could inflate the family-history association. The absence of objective measures (HbA1c, lipid levels, blood pressure) prevented dose–response analyses, and height and weight were self-reported. The CVD item did not distinguish coronary heart disease, stroke, heart failure, or other conditions, so the prevalence and overlap of subtypes could not be reported and subtype-specific analyses were not possible. The number of CVD events (n = 41) was small relative to the number of candidate variables, producing wide confidence intervals; penalised estimation and a reduced model were used to address this, but the estimates remain imprecise and should be regarded as exploratory. Finally, participants were recruited by convenience sampling within purposively selected sites in a single region, which limits generalisability. Future prospective studies with validated endpoints and objective metabolic phenotyping are needed.

## 5. Conclusions

In this exploratory analysis of adults with T2DM in the Jazan region, a positive family history of CVD, diagnosed hypertension, and diagnosed dyslipidaemia were strongly associated with prevalent self-reported cardiovascular disease, and regular whole-grain consumption was associated with lower odds of CVD. Physical inactivity, smoking, and BMI were not significantly associated with CVD after adjustment; given the single-item measures and small number of events, these null findings do not indicate that these factors lack cardiovascular relevance. Because the cross-sectional design cannot establish temporality, the findings describe associations rather than causes. They support documenting family history of CVD and ensuring guideline-directed blood pressure and lipid management in patients with diabetes, and they identify whole-grain intake as a dietary factor worth testing in prospective studies.

## Figures and Tables

**Table 1:** Sociodemographic characteristics of the study population (n = 423).

Variable	n	%
**Age (years), mean ± SD**	**48 ± 15**	
**Sex**		
Male	252	60%
Female	171	40%
**Nationality**		
Saudi	414	98%
Non-Saudi	9	2%
**Education**		
Bachelor’s degree or higher	225	53%
High school or below	198	47%
**Employment status**		
Employed	261	62%
Unemployed	162	38%
**Marital status**		
Married	309	73%
Unmarried	114	27%
**Monthly income (SAR)**		
<5000	114	27%
5000–9999	73	17%
10,000–14,999	112	26%
≥15,000	124	30%
**Residence**		
Rural	268	63%
Urban	155	37%
**Housing type**		
Owned villa	192	45%
Owned traditional house	116	27%
Owned apartment	83	20%
Rented	32	8%
**Family size, mean ± SD**	**7.3 ± 2.9**	

**Table 2:** Habitual and health-related characteristics (n = 423).

Variable	n	%
**BMI (kg/m^2^), mean ± SD**	**27.2 ± 5.4**	
**BMI category**		
Overweight (25.0–29.9)	161	38%
Normal weight (18.5–24.9)	135	32%
Obese (≥30.0)	112	26%
Underweight (<18.5)	15	4%
**Physical activity**		
No weekly physical activity	180	43%
Moderate/vigorous < 30 min, 5×/week	165	39%
Moderate/vigorous ≥ 30 min, 5×/week	78	18%
**Smoking status**		
Never smoker	331	78%
Current smoker	50	12%
Ex-smoker	42	10%
**Sleep duration (hours), mean ± SD**	**7.3 ± 1.8**	
**Family history of diabetes mellitus**	365	86%
**Family history of hypertension**	246	58%
**Family history of CVD**	85	20%
**Diagnosed hypertension**	202	48%
**Diagnosed dyslipidemia**	44	10%
**Diagnosed CVD**	41	10%

**Table 3:** Nutritional characteristics (n = 423).

Variable	n	%
**Whole grain product consumption**		
No	224	53%
Yes	199	47%
**≥5 servings of fruits and vegetables per day**		
No	312	74%
Yes	111	26%
**Chooses low-fat meats**		
Yes	255	60%
No	168	40%
**Avoids foods high in sugar**		
No	230	54%
Yes	193	46%
**Chooses low-fat products**		
No	264	62%
Yes	159	38%

**Table 4:** Bivariate associations with CVD diagnosis (n = 423).

Variable	No CVD (n = 382)	CVD (n = 41)	*p*-Value
Age, mean ± SD	47 ± 15	50 ± 18	0.233
**Sex**			0.327
Female	151 (40%)	20 (49%)	
Male	231 (60%)	21 (51%)	
**Education**			0.577
Bachelor’s or higher	201 (53%)	24 (59%)	
High school or below	181 (47%)	17 (41%)	
**Employment**			0.344
Employed	239 (63%)	22 (54%)	
Unemployed	143 (37%)	19 (46%)	
**Marital status**			0.868
Married	280 (73%)	29 (71%)	
Unmarried	102 (27%)	12 (29%)	
**Income**			0.582
<5000 SAR	101 (26%)	13 (32%)	
5000–9999 SAR	69 (18%)	4 (10%)	
10,000–14,999 SAR	101 (26%)	11 (27%)	
≥15,000 SAR	111 (29%)	13 (32%)	
**Residence**			0.127
Rural	247 (65%)	21 (51%)	
Urban	135 (35%)	20 (49%)	
Family size, mean ± SD	7.3 ± 2.8	7.6 ± 3.3	0.434
BMI, mean ± SD	27 ± 5.3	28 ± 5.9	0.500
**BMI category**			0.422
Underweight	13 (3%)	2 (5%)	
Normal weight	121 (32%)	14 (34%)	
Overweight	150 (39%)	11 (27%)	
Obese	98 (26%)	14 (34%)	
**Smoking status**			**0.048**
Never smoker	300 (79%)	31 (76%)	
Current smoker	48 (13%)	2 (5%)	
Ex-smoker	34 (9%)	8 (20%)	
**Whole grain consumption**			**0.025**
No	195 (51%)	29 (71%)	
Yes	187 (49%)	12 (29%)	
**≥5 fruit/vegetable servings/day**			0.515
No	284 (74%)	28 (68%)	
Yes	98 (26%)	13 (32%)	
**Low-fat meat choice**			0.549
No	154 (40%)	14 (34%)	
Yes	228 (60%)	27 (66%)	
**Avoidance of sugary foods**			0.554
No	210 (55%)	20 (49%)	
Yes	172 (45%)	21 (51%)	
**Low-fat product choice**			0.976
No	239 (63%)	25 (61%)	
Yes	143 (37%)	16 (39%)	
**Physical activity**			0.775
No activity	161 (42%)	19 (46%)	
<30 min, 5×/week	149 (39%)	16 (39%)	
≥30 min, 5×/week	72 (19%)	6 (15%)	
**Family history of DM**			0.311
No	55 (14%)	3 (7%)	
Yes	327 (86%)	38 (93%)	
**Family history of HTN**			**0.011**
No	168 (44%)	9 (22%)	
Yes	214 (56%)	32 (78%)	
**Family history of CVD**			**<0.001**
No	325 (85%)	13 (32%)	
Yes	57 (15%)	28 (68%)	
**Dyslipidemia diagnosis**			**<0.001**
No	349 (91%)	30 (73%)	
Yes	33 (9%)	11 (27%)	
**Hypertension diagnosis**			**<0.001**
No	214 (56%)	7 (17%)	
Yes	168 (44%)	34 (83%)	

Bold *p*-values indicate statistical significance at *p* < 0.05.

**Table 5:** Full multivariable models of factors associated with prevalent CVD among participants with diabetes.

Variable	Reference	Standard Logistic Regression			Firth Penalised Regression		
		OR	95% CI	*p*	OR	95% CI	*p*
**Sociodemographic**							
Age (per year)	*—*	1.00	0.97–1.04	0.890	1.00	0.97–1.03	0.893
Sex: male	*Female*	0.63	0.20–1.90	0.414	0.69	0.25–1.87	0.467
Nationality: non-Saudi	*Saudi*	5.53	0.21–68.07	0.215	4.80	0.38–39.11	0.198
Education: bachelor’s or higher	*High school or below*	1.12	0.39–3.26	0.836	1.09	0.42–2.86	0.865
Employment: employed	*Unemployed*	0.51	0.18–1.43	0.203	0.57	0.22–1.43	0.231
Marital status: married	*Unmarried*	1.05	0.34–3.34	0.930	1.03	0.38–2.91	0.954
Income: 5000–9999 SAR	*<5000 SAR*	0.16	0.03–0.72	**0.024**	0.23	0.05–0.89	**0.032**
Income: 10,000–14,999 SAR	*<5000 SAR*	0.28	0.06–1.09	0.075	0.36	0.10–1.21	0.100
Income: ≥15,000 SAR	*<5000 SAR*	0.36	0.08–1.62	0.190	0.43	0.11–1.67	0.226
Residence: urban	*Rural*	1.81	0.72–4.63	0.210	1.67	0.73–3.86	0.227
Housing: owned apartment	*Rented*	1.01	0.17–7.46	0.995	0.94	0.19–5.40	0.941
Housing: owned traditional house	*Rented*	0.34	0.05–2.71	0.285	0.38	0.07–2.35	0.287
Housing: owned villa	*Rented*	0.40	0.06–3.01	0.344	0.43	0.09–2.51	0.330
Family size (per person)	*—*	1.08	0.91–1.28	0.395	1.06	0.91–1.24	0.444
**Anthropometric and behavioural**							
BMI (per kg/m^2^)	*—*	0.95	0.87–1.04	0.283	0.96	0.89–1.04	0.322
Physical activity: <30 min, 5×/week	*No activity*	0.96	0.34–2.71	0.933	0.96	0.38–2.45	0.930
Physical activity: ≥30 min, 5×/week	*No activity*	0.61	0.13–2.57	0.508	0.68	0.17–2.46	0.565
Smoking: current smoker	*Never smoker*	0.93	0.12–4.97	0.936	1.01	0.17–4.36	0.988
Smoking: ex-smoker	*Never smoker*	1.30	0.32–5.02	0.709	1.27	0.36–4.22	0.698
**Nutritional**							
Whole grain consumption	*No*	0.23	0.07–0.64	**0.007**	0.30	0.11–0.75	**0.010**
≥5 fruit/vegetable servings/day	*No*	1.03	0.38–2.67	0.947	1.04	0.42–2.44	0.924
Low-fat meat choice	*No*	1.57	0.56–4.63	0.399	1.45	0.57–3.84	0.441
Avoidance of sugary foods	*No*	1.61	0.63–4.27	0.328	1.46	0.63–3.49	0.383
Low-fat product choice	*No*	0.80	0.30–2.03	0.645	0.84	0.35–1.93	0.681
**Clinical and hereditary**							
Family history of DM	*No*	2.23	0.53–12.19	0.304	1.77	0.50–7.74	0.393
Family history of HTN	*No*	0.94	0.30–3.01	0.915	0.93	0.34–2.65	0.889
Family history of CVD	*No*	25.66	9.03–85.31	**<0.001**	14.57	5.88–40.90	**<0.001**
Dyslipidemia diagnosis	*No*	7.21	2.35–22.75	**<0.001**	5.19	1.91–14.22	**0.001**
Hypertension diagnosis	*No*	6.59	2.32–21.11	**<0.001**	4.71	1.86–13.16	**<0.001**
**Observations**		423			423		
**Tjur R^2^**		0.362			0.352		

OR = odds ratio; CI = confidence interval; BMI = body mass index; CVD = cardiovascular disease; DM = diabetes mellitus; HTN = hypertension; SAR = Saudi Arabian Riyal. Standard model: profile likelihood 95% CIs and Wald *p*-values. Firth model: profile penalised-likelihood 95% CIs and penalised likelihood ratio *p*-values. n = 423 (41 CVD events); 29 model parameters, all shown. Bold *p*-values indicate *p* < 0.05.

**Table 6:** Reduced multivariable models restricted to established cardiovascular risk factors among participants with diabetes.

Variable	Reference	Standard Logistic Regression			Firth Penalised Regression		
		OR	95% CI	*p*	OR	95% CI	*p*
**Sociodemographic**							
Age (per year)	*—*	0.99	0.97–1.02	0.593	0.99	0.97–1.02	0.589
Sex: male	*Female*	0.93	0.38–2.27	0.874	0.94	0.40–2.23	0.893
**Anthropometric and behavioural**							
BMI (per kg/m^2^)	*—*	0.96	0.89–1.03	0.277	0.96	0.89–1.03	0.294
Physical activity: <30 min, 5×/week	*No activity*	0.77	0.32–1.81	0.550	0.78	0.34–1.78	0.558
Physical activity: ≥30 min, 5×/week	*No activity*	0.62	0.17–1.99	0.437	0.65	0.19–1.99	0.462
Smoking: current smoker	*Never smoker*	0.45	0.06–2.00	0.350	0.54	0.09–2.14	0.405
Smoking: ex-smoker	*Never smoker*	1.05	0.30–3.46	0.935	1.07	0.32–3.39	0.905
**Clinical and hereditary**							
Family history of CVD	*No*	16.60	7.43–39.98	**<0.001**	14.04	6.51–32.41	**<0.001**
Dyslipidemia diagnosis	*No*	3.30	1.24–8.53	**0.015**	3.17	1.23–7.89	**0.018**
Hypertension diagnosis	*No*	7.37	2.99–20.64	**<0.001**	6.41	2.71–17.02	**<0.001**
**Observations**		423			423		
**Tjur R^2^**		0.286			0.283		

OR = odds ratio; CI = confidence interval; BMI = body mass index; CVD = cardiovascular disease; DM = diabetes mellitus; HTN = hypertension; SAR = Saudi Arabian Riyal. Standard model: profile likelihood 95% CIs and Wald *p*-values. Firth model: profile penalised-likelihood 95% CIs and penalised likelihood ratio *p*-values. n = 423 (41 CVD events); 10 model parameters. Bold *p*-values indicate *p* < 0.05.

## Data Availability

The data presented in this study is available on request from the corresponding author. The data are not publicly available due to ethical restrictions and privacy concerns related to sensitive patient health information.

## Abbreviations

The following abbreviations are used in this manuscript:

CVD	Cardiovascular disease
T2DM	Type 2 diabetes mellitus
HTN	Hypertension
BMI	Body Mass Index
STEPS	STEPwise Approach to Surveillance
IPAQ	International Physical Activity Questionnaire
